# Prediction of Current and Future Distributions of *Chalcophora detrita* (Coleoptera: Buprestidae) Under Climate Change Scenarios

**DOI:** 10.1002/ece3.70693

**Published:** 2025-01-16

**Authors:** Arif Duyar, Muhammed Arif Demir, Mahmut Kabalak

**Affiliations:** ^1^ Department of Applied Biology, Graduate School of Science and Engineering Hacettepe University Ankara Türkiye; ^2^ The Scientific and Technological Research Council of Türkiye (TUBITAK) Ankara Türkiye; ^3^ Biology Department, Faculty of Science Hacettepe University Ankara Türkiye; ^4^ Biological Diversity Research and Application Center Hacettepe University Ankara Türkiye

**Keywords:** Buprestidae, *Chalcophora detrita*, climate change, ecological niche modelling, Mediterranean biodiversity, saproxylic beetles

## Abstract

The consequences of climate change, accelerated by anthropogenic activities, have different effects on different ecosystems, and the severity of these effects is predicted to increase in the near future. The number of studies investigating how forest ecosystems respond to these changes is increasing. However, there remains a significant gap in research concerning how saproxylic organisms—one of the key contributors to the healthy functioning of these fragile ecosystems—will respond to the consequences of climate change. In our study, we estimated the suitable habitats of the polymorphic species *Chalcophora detrita* which is distributed across Italy, Albania, Bulgaria, Greece, Türkiye, Cyprus, Syria, Israel and Lebanon. This species of both saproxylic and economic importance, was modelled under current environmental conditions, climate change scenarios and possible future conditions by ecological niche modelling (ENM). An ensemble model was created by using 11 different algorithms (Artificial Neural Network, Classification Tree Analysis, eXtreme Gradient Boosting, Flexible Discriminant Analysis, Generalised Additive Model, Generalised Boosting Model, Generalised Linear Model, Multivariate Adaptive Regression Splines, Maximum Entropy, Random Forest, Surface Range Envelope) to predict the potential suitable habitats of *C. detrita*. Two different future scenarios (SSP2‐4.5, relatively optimistic and SSP5‐8.5, most pessimistic) are divided into 2021–2040, 2041–2060, 2061–2080 and 2081–2100 time periods. The results of our ENM indicated that bioclimatic variables contribute more than topographic and land cover variables to suitable habitats for the species under current conditions. Furthermore, future scenarios demonstrated that suitable habitats for this species will gradually decrease across the geographical region where the species is distributed. This study provides a theoretical reference framework for the conservation of habitats and the improvement of management plans for species belonging to the genus *Chalcophora* Dejean 1833 and the other saproxylic beetles.

## Graphical abstract

This study provides a theoretical reference framework for the conservation of habitats and the improvement of management plans for species belonging to the genus Chalcophora Dejean 1833 and the other saproxylic beetles. On the other hand, we believe that the ecological roles of Chalcophora species should be re‐evaluated and ecological niche modelling studies should be considered together with biotic factors.

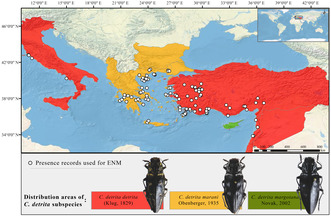

## 1 Introduction

The effects of climate change are observed not only at the organism level but also at the ecosystem level, as they are intricately linked to organisms undergoing biological changes. Forests are one of these delicate ecosystems. The possible consequences of climate change on forests and forest invertebrates are affected not only by the increase in temperature, but also by multiple factors such as the increase in atmospheric CO_2_, the frequency of extreme droughts, and changes in humidity (Jactel, Koricheva, and Castagneyrol 2019). Even the tree species have evolved to withstand seasonal water scarcity as in Mediterranean forests may struggle to cope with future increases in drought frequency and intensity due to anthropogenic influences. Considering the change in temperatures and the increase in the frequency of dry periods, it is also predicted that there will be significant changes in the behaviour, biology and ecology of insects that can colonise bark and wood (Salle, Nageleisen, and Lieutier 2014). There are known cases where xylophagous species attacking weakened trees can colonise healthy trees and become primary pests (Balla et al. 2021; Valenta et al. 2016).

Commonly known as jewel beetles, Buprestidae (Insecta: Coleoptera) are distributed across all continents except Antarctica, with over 15,000 species (Bellamy and Volkovitsh 2016). In the Palaearctic region, it is represented by 2629 species (Löbl and Löbl 2016). The larvae of this family develop within plant tissues, and some of them are leaf miners, while others induce gall formation on limbs and twigs, and some bore through plant roots. However, the majority of them bore into the wood or bark of living or dead trees (Nelson et al. 2008).

*Chalcophora* Dejean 1833 (Chrysochroinae: Chalcophorini) is a genus that is widely distributed and comprising relatively large species with economic importance (Maier and Ivie 2013) and also with saproxylic roles for the forest ecosystems. There are currently 15 valid species belonging to this genus worldwide (Bellamy 2008). The majority of *Chalcophora* species have similar life cycles. Their larvae bore into the underside of living pine tree (*Pinus* spp.) bark, excavating long, flat and winding tunnels. They continue to excavate towards the heartwood to enter the pupal stage and emerge as adults in either spring or autumn (Maier 2010) and early summer (Lundberg 1983).

Apart from their economic importance, the genus *Chalcophora*, which consists of saproxylic species, is also important in terms of its role in the forest ecosystem. A balanced presence of organisms that can colonise decaying or dead trees, stumps, green or dead leaves, fresh or rotting fruit, and debris in tree hollows is essential for healthy tree populations (Carpaneto et al. 2015). Although the exact number of saproxylic species is unknown, according to expert opinion there may be nearly 4000 saproxylic insect species in Europe (Cálix et al. 2018). The current IUCN European Red List has provided an assessment for some of the saproxylic insect species. In 2008, and additionally in 2017, a total of 693 species were evaluated. In Europe, as a general trend, approximately 17.9% of species are considered to be under threat (Cálix et al. 2018). While various factors contribute to these threats, temperature and precipitation changes stemming from anthropogenic activities lead to reductions in suitable habitats for wild species, causing these species to shift towards sub‐optimal conditions or face extinction (Parmesan 2006).

In this study, we used ecological niche modelling (ENM) to assess the future of *Chalcophora detrita* under climate change by predicting the extent of current and future suitable areas. We test whether this species will show one or more of the following responses: (1) reduction of the suitable areas, (2) future shift of suitable areas towards northern latitudes and (3) localised habitat loss leading to subspecies extinction.

## 2 Materials and Methods

### 2.1 Taxonomic Framework and Distribution of Examined Species

The polymorphic species *Chalcophora detrita* (Klug 1829) is a member of the genus *Chalcophora* Dejean 1833 in the tribe Chalcophorini (Buprestidae: Chrysochroinae). This species is represented by three subspecies: *C. detrita detrita* (Klug 1829), *C. detrita marani* Obenberger 1935, and *C. detrita margotana* Novak 2002. Although, *C. detrita detrita* is distributed in Italy, Greece (Aegean Islands), Israel, Lebanon, Syria and Türkiye (Anatolia); *C. detrita marani* is distributed in Albania, Bulgaria, Greece, and Türkiye (Thrace). *C. detrita margotana* is an endemic subspecies for Cyprus (Kubáň 2016) (Figure 2). Here, the absence of records of *C. detrita detrita* in Greece while it is distributed in Türkiye and Italy may initially seem contradictory and controversial, but it is believed that this subspecies was introduced to Southern Italy from the Middle East through trade routes between 1000 and 2000 years ago (Ruzzier et al. 2023).

The morphological differences of these three subspecies are as follows: The pronotal central keel of *C. detrita detrita* is narrower and somewhat raised and the five flattened elytral costae on the rear half of the elytra are only partially present. The five elytral costae of *C. detrita marani* are clearly elevated and also clearly visible on the rear half of the elytra. The pronotal central keel of *C. detrita margotana* is broader, the elytral costae are flattened and missing laterally on the rear half of the elytra (Novak 2002).

**FIGURE 1:**
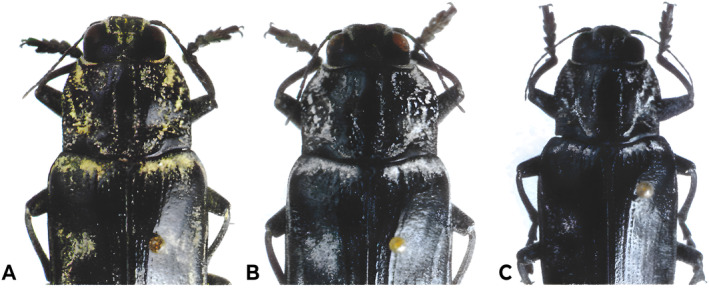
Morphological characters of *Chalcophora detrita* subspecies. (A) *Chalcophora detrita detrita* (Klug 1829). (B) *Chalcophora detrita marani* Odenberger 1935. (C) *Chalchophora detrita margotana* Novak (2002). Photographs were adopted from Novak (2002).

According to Novak (2002), the differently coloured dusting of the specimens could be used as a taxonomic character for *Chalcophora detrita* subspecies identification. The basic form of *C. detrita detrita* is heavily yellow‐dusted. However, the *C. detrita marani* exhibits snow‐white dusting. Novak has shown that carefully treated specimens can be immediately assigned based on the dusting, nevertheless, we think that this type of identification is controversial due to the difficulty of preserving samples. As Novak (2002) mentioned in his study these large insects are almost exclusively killed in ethyl acetate ether or alcohol‐soaked substrate. In this process, the dusting is scraped off because these large insects dig around in the container for a longer time until the ethyl acetate ether or alcohol intoxicates them (Figure 1).

### 2.2 The Study Area and the Presence Data

In this study, the records for these three subspecies were evaluated together as they were indistinguishable, and the study area was limited to the area where the three subspecies were distributed (32°–48° N, 10°–44° E).

Presence data for the species used in ecological niche models was obtained from the field studies conducted in the Inner West Anatolian part of Türkiye (Afyonkarahisar, Kütahya, Uşak provinces and the northern part of Denizli province) in 2019, 2021 and 2022 (52 records), databases (GBIF—207 records, iNaturalist—153 records, Observation—2 records) and literature (Kanat 2001; Gürkan 2011; https://virtualcollections.naturalsciences.be (1 record each)).

A total of 417 occurrence records were subjected to spatial and environmental dilution to reduce the effects of spatial sampling biases and to obtain a more homogeneous distribution of occurrence records within the distribution area. For spatial dilution, the ‘spThin’ package version 0.2.0 (Aiello‐Lammens et al. 2015) in R version 4.2.3 (R Core Team 2023) was used to filter records at a distance of 5 km and diluted by eliminating multiple records, resulting in 115 records. Environmental filtering was performed with five bins using environmental variables (variable set 4, see Environmental variable) through the ‘occfilt_env’ function in the ‘flexSDM’ package version 1.3.4 (Velazco et al. 2022) in R version 4.2.3 (R Core Team 2023). This method basically reduces the environmental redundancy in your data and is based on the methods summarised by Varela et al. (2014). As a result, 107 records were included in the models.

### 2.3 Environmental Variable

Current and future bioclimatic data were obtained from the WorldClim database version 2.1 (https://www.worldclim.org/data/worldclim21.html), with a spatial resolution of 30 arc‐seconds (Fick and Hijmans 2017). Nineteen scenopoetic bioclimatic variables were derived from monthly temperature and precipitation values (Peterson et al. 2011). Future bioclimatic data from the Coupled Model Intercomparison Project Phase 6 (CMIP6) (Eyring et al. 2016) are based on one global climate model (MIROC6), two Shared Socio‐economic Pathways (SSP2‐4.5 and SSP5‐8.5) and four time periods (2021–2040, 2041–2060, 2061–2080 and 2081–2100) in this study. The assessed best estimates and very likely ranges of warming for 2081–2100 relative to 1850–1900 vary from 2.7°C [2.1°C—3.5°C] in the intermediate GHG emissions scenario (SSP2‐4.5) to 4.4°C [3.3°C—5.7°C] in the very high GHG emissions scenario (SSP5‐8.5) (IPCC 2021). For GHG emissions, SSP2‐4.5 projects a future where GHG emissions peak around 2040 and start to decline. In contrast, SSP5‐8.5 projects a future where GHG emissions continue to increase throughout the century (Riahi et al. 2017). In this study, four of the 19 bioclimatic variables (BIO8, BIO9, BIO18 and BIO 19) were excluded because of artificial discontinuities in the climate gradients.

The topography variables were obtained at a resolution of 30 arc‐seconds (~1 km) from the EarthEnv dataset (https://www.earthenv.org/topography) (Amatulli et al. 2018). These variables are derived based on SRTM4.1dev products at 90‐m resolution on a nearly global scale. This study used five variables (elevation, slope, roughness, topographic position index and terrain ruggedness index).

The land cover variables were obtained at 100‐m resolution by Copernicus Global Land Monitoring Service from the Copernicus repository (https://land.copernicus.eu/global/products/lc). This dataset provides a discrete classification with 23 classes (six types closed forest, six types open forest, Shrub, Herbaceous vegetation, Herbaceous wetland, Moss and lichen, Bare/sparse vegetation, Cultivated and managed vegetation (cropland), Urban/Built‐up, Snow and Ice, Permanent inland water bodies, Missing data, Open Sea) based on the UN‐FAO Land Cover Classification System (Buchhorn et al. 2020). In this study, only forest variable was used since it is thought to affect the distribution of the species.

All raster data for the variables were converted to resolution of 30 arc‐seconds (~1 km) and aligned. To reduce the overfitting of the ENM, highly correlated variables were removed based on the variance inflation factor (VIF), which is a precise method and measures how strongly each predictor can be explained by other predictors. The VIF is based on the square of the multiple correlations arising from the regression of the predictor variable against all other predictors (Naimi and Araújo 2016). Multicollinearity between these variables was calculated using the VIF (Lin, Foster, and Ungar 2011) with the ‘usdm’ package version 2.1–7 (Naimi 2017) and the ‘vifstep’ function (threshold = 10) in R software version 4.2.3 (R Core Team 2023) (Table 1). The potential habitats of *C. detrita* under current conditions were predicted using four separate sets of variables (Set 1: bioclimatic variables, Set 2: bioclimatic and topographic variables, Set 3: bioclimatic and land cover variables, and Set 4: bioclimatic, topographic and land cover variables) (Table 2) and future conditions were predicted using a single data set (Set 2) consisting of bioclimatic and topographic variables (Table 1).

**TABLE 1:** Variable sets used for prediction of suitable habitats of *C. detrita* under current conditions (variables selected by VIF analysis (threshold = 10) for use in the models are highlighted with yellow along with their final VIF values, variables indicated with ‘+’ have VIF values > 10).

Code	Variables	Set 1	Set 2	Set 3	Set 4
Bioclimatic variables					
BIO1	Annual mean temperature (°C)	+	+	+	+
BIO2	Mean diurnal range (°C)	+	+	+	+
BIO3	Isothermality (%) (100 * BIO2/BIO7)	13,588	30,124	13,600	31,375
BIO4	Temperature seasonality (100 * SD)	14,240	55,824	14,146	54,810
BIO5	Max temperature of warmest month (°C)	14,196	76,861	14,295	80,404
BIO6	Min temperature of coldest month (°C)	+	+	+	+
BIO7	Temperature annual range (°C) (BIO5—BIO6)	+	+	+	+
BIO10	Mean temperature of warmest quarter (°C)	+	+	+	+
BIO11	Mean temperature of coldest quarter (°C)	+	+	+	+
BIO12	Annual precipitation (mm)	15,549	19,577	16,535	20,109
BIO13	Precipitation of wettest month (mm)	+	+	+	+
BIO14	Precipitation of driest month (mm)	16,517	32,322	16,981	32,120
BIO15	Precipitation seasonality (coefficient of variation)	+	+	+	+
BIO16	Precipitation of wettest quarter (mm)	+	+	+	+
BIO17	Precipitation of driest quarter (mm)	+	+	+	+
Topographic variables					
—	Elevation (m)	−	94,506	−	96,197
—	Slope	−	16,713	−	19,218
—	Roughness	−	+	−	+
TPI	Topographic position index	−	11,160	−	11,359
TRI	Terrain ruggedness index	−	+	−	+
Land cover variables					
—	Forest (%)	−	−	11,324	15,456

**TABLE 2:** Percentages of gain–loss and stability of suitable habitats in the future scenarios compared with the current prediction.

	Gain (pixel)	Loss (pixel)	Stable suitable (pixel)	Stable unsuitable (pixel)	Gain (%)	Loss (%)	Species range change (%)
Current—SSP245 2021–2040	40,224	60,857	395,436	4,181,720	8.82	13.34	−4.52
Current—SSP245 2041–2060	49,512	106,636	349,657	4,172,432	10.85	23.37	−12.52
Current—SSP245 2061–2080	49,242	157,173	299,120	4,172,702	10.79	34.45	−23.65
Current—SSP245 2081–2100	52,472	192,850	263,443	4,169,472	11.50	42.27	−30.77
Current—SSP585 2021–2040	39,103	65,007	391,286	4,182,841	8.57	14.25	−5.68
Current—SSP585 2041–2060	46,084	164,307	291,986	4,175,860	10.10	36.01	−25.91
Current—SSP585 2061–2080	49,187	251,419	204,874	4,172,757	10.78	55.10	−44.32
Current—SSP585 2081–2100	36,960	316,349	139,944	4,184,984	8.10	69.33	−61.23

### 2.4 Ecological Niche Modelling (ENM)

To predict the potential suitable habitats of *C. detrita*, an ensemble model was created by using 11 different algorithms (Artificial Neural Network—ANN, Classification Tree Analysis—CTA, eXtreme Gradient Boosting—XGBOOST, Flexible Discriminant Analysis—FDA, Generalised Additive Model—GAM, Generalised Boosting Model—GBM, Generalised Linear Model—GLM, Multivariate Adaptive Regression Splines—MARS, Maximum Entropy—MAXENT, Random Forest—RF, Surface Range Envelope—SRE/BIOCLIM) with the ‘biomod2’ package version 4.2.4 (Thuiller et al. 2023) in R version 4.2.3 (R Core Team 2023). The ‘ensemble model’ is a proportional combination of responses of the algorithms. It exploits the advantages of these algorithms while minimising the disadvantages of each one (Araújo and New 2007; Thuiller et al. 2009, 2016), improves accuracy and provides a measure of uncertainty in predictions (Araújo and New 2007).

The CTA, XGBOOST, FDA, GAM, GBM, GLM, MARS and RF algorithms utilised in this study require simulated pseudo‐absence data in addition to presence records to train and test the models. Barbet‐Massin et al. (2012) and Wisz and Guisan (2009) indicated that, particularly for regression‐based techniques (e.g., GLM, GAM and MARS), the random method provides higher model accuracy compared to selection methods like ‘SRE’ or ‘disk.’ For classification and machine‐learning techniques, while the pseudo‐absence data generation method has less impact on predictive accuracy, the ‘2° far’ method yielded better models with fewer presence points, and the ‘SRE’ method performed better with a higher number of presence points (Barbet‐Massin et al. 2012). Since the majority of presence records were obtained from online databases, these records may not fully capture the species' geographical or environmental distribution. This leads to a presence sample that does not encompass the species' entire realised niche. To mitigate this sampling bias, the ‘disk’ or ‘SRE’ methods were not used for generating pseudo‐absence data. Instead, the ‘random’ method was chosen, resulting in the generation of 1000 pseudo‐absence points (for a detailed description of pseudo‐absence data selection strategies, see Thuiller et al. 2023). The random method selects pseudo‐absence points within the study area without geographical or environmental assumptions, aiming to reduce sampling bias. Although the random method could introduce potential biases of its own, these were mitigated by generating four distinct sets of 1000 pseudo‐absence points, with repeated sampling for each algorithm. This process aimed to expand model training coverage and improve robustness. Additionally, the presence and pseudo‐absence data were weighted to ensure a neutral prevalence of 0.5. The algorithms were fitted using the default settings in the ‘biomod2’ package, with some modifications based on suggestions by Valavi et al. (2022). For further details on the algorithms, please refer to Elith et al. (2006) and Valavi et al. (2022).

The MAXENT algorithm can be applied using presence‐background data (Phillips, Anderson, and Schapire 2006). The model settings were chosen using the ‘ENMeval’ package version 2.0.4 (Kass et al. 2021) in R version 4.2.3. A customised hypothesis regarding the accessible area for Maxent model calibration has been established (M in the BAM diagram; Soberon and Peterson 2005), which is most appropriate as a delimitation of an area for model calibration (Barve et al. 2011). The BAM framework provides a theoretical basis for understanding scenarios of how biotic (B), abiotic (A) and dispersal (M, mobility) factors determine species distributions (Soberon and Peterson 2005). The M hypotheses were based on dispersion simulations using the ‘grinnell’ package version 0.0.22 (Machado‐Stredel, Cobos, and Peterson 2021). We tested different values for two parameters: the number of dispersal events (50, 100, 150, 200, 250, 300, 350 and 400) and the dispersion kernel standard deviation (1, 2, and 3). (Dispersal kernel = ‘normal’, suitability threshold = %5, Maximum number of dispersers = 4). Simulations were carried out using the variables in variable set 4 under the current environmental conditions. The selection of the calibration area was based on an analysis of each simulated area, which was evaluated in accordance with the known distribution of the species and the known biogeographical ranges (Appendix S2). In addition, 50,000 pixels of background data were obtained from each simulated area and models were created. The best model was determined according to the area under the curve (AUC) values and evaluated by projecting to the current. According to the determined accessible area (area M), candidate models were created with the ENMeval package for each set of variables for optimisation in the biomod2 package of the Maxent algorithm. In the Maxent algorithm, model complexity can be controlled by a set of parameters called feature classes (FCs) and regularisation multiplier (RM). FCs aim to improve model fit (Elith et al. 2011) and consist of a transformation of the original predictor variables [i.e., linear (L), quadratic (Q), hinge (H), product (P) and threshold (T)] that can be used separately or together, while RM aims to reduce overfitting (Merow, Smith, and Silander Jr 2013). The fit of 250 different candidate models was determined by considering 5 FC combinations (L, LQ, H, LQH and LQHP) and RM values ranging from 0.1 to 5, changing every 0.1 step. The model settings were selected based on the model with the highest value of the mean AUC test (Appendix S3).

In the ensemble model (and also in the maxent candidate models), a spatial partition (block, *k* = 4) was used to divide the presence and background/pseudo‐absence data into four separate training and test boxes. Four evaluation runs were performed, yielding a total of 176 single models, whose performance was evaluated using the AUC of receiver operator characteristics (ROC) (Phillips, Anderson, and Schapire 2006). Individual models with AUC > 0.8 were selected for ensemble modelling. The ‘wmean’ algorithm (weighted average of probabilities) was used to build the ensemble model. This is a procedure where each model is assigned a score proportionally according to its performance during ensemble building. The application of spatial partitioning (block method) is of considerable importance in the reduction of potential biases, achieved through the diminution of spatial autocorrelation in both the presence and pseudo‐absence data. By dividing the data into distinct spatial blocks, each model is trained and tested on spatially independent data, thereby limiting the potential for localised spatial biases to affect model predictions. This approach enhances the robustness of each individual model and ensures that the ensemble model is less susceptible to overfitting to spatial clusters. Moreover, the use of ensemble modelling contributes to the reduction of bias by combining multiple models, each with distinct patterns and assumptions. The ‘wmean’ method prioritises models with higher predictive performance, which helps to mitigate the influence of weaker models and further reduces the impact of individual model biases. However, it is important to acknowledge that while ensemble modelling and spatial partitioning can substantially reduce biases, they may not eliminate them entirely, especially if multiple models share underlying assumptions or data limitations. Nevertheless, the ensemble approach remains an effective strategy to leverage the strengths of various models while minimising their individual weaknesses. The model outputs were organised using ArcMap 10.8.2 (ESRI 2021).

## 3 Results

### 3.1 Variable Contributions to Suitable Habitats

**FIGURE 2:**
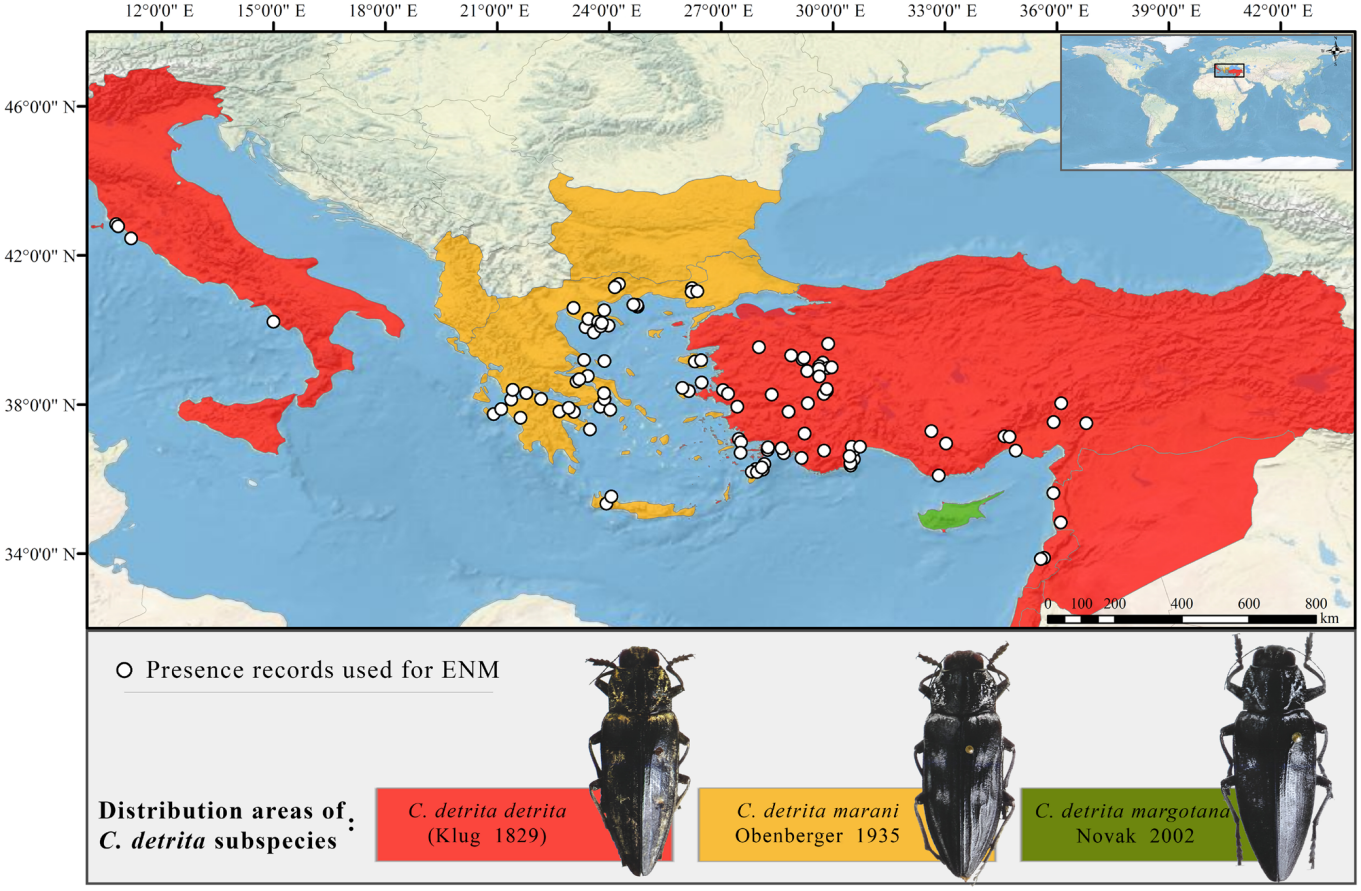
Map of presence records used in the models. The country‐based distribution data of the subspecies were obtained from the Kubáň (2016) and subspecies photographs were adopted from Novak (2002).

**FIGURE 3:**
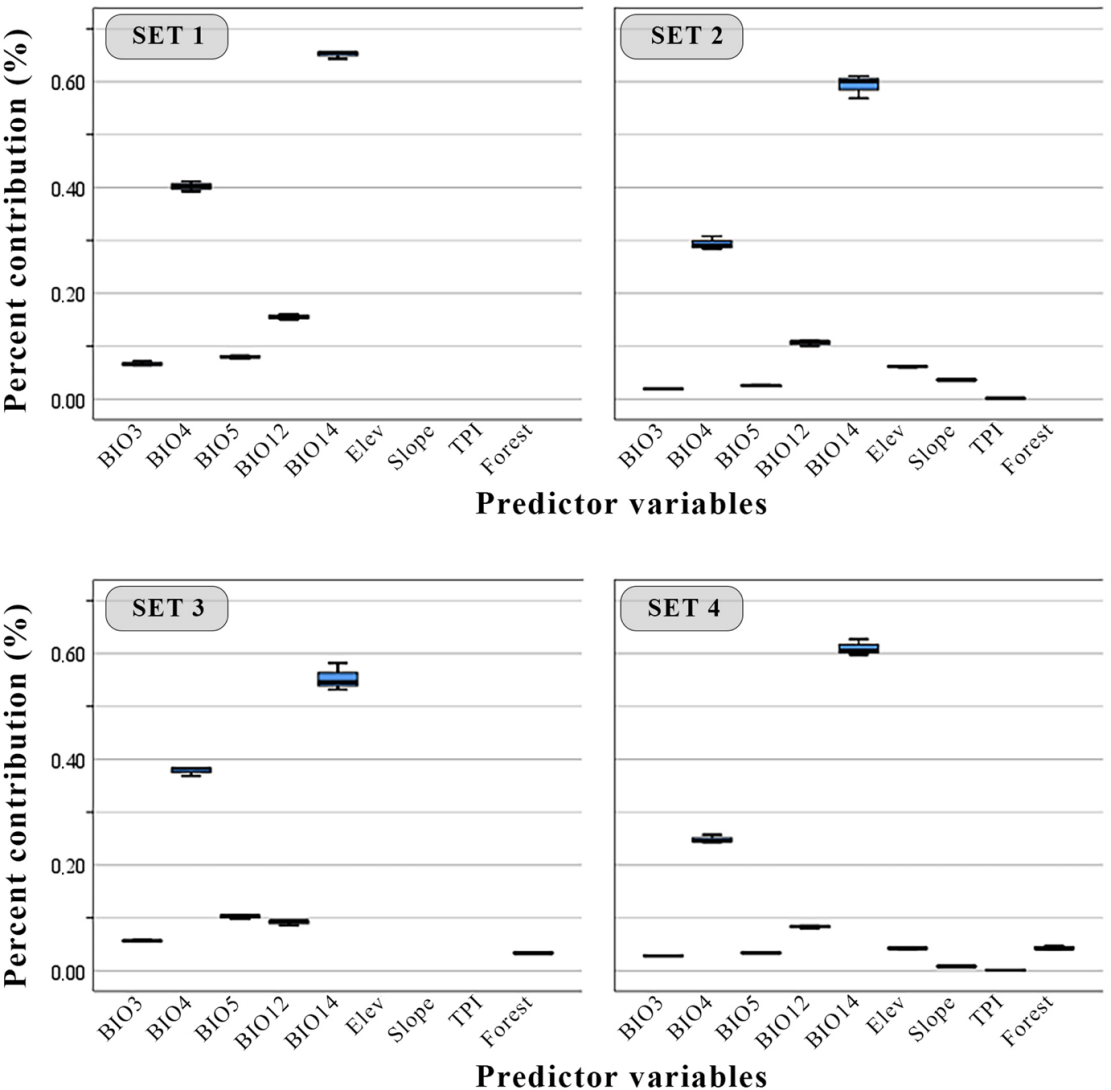
Main variables and contribution percentages used in the four ensemble models. BIO12, annual precipitation; BIO14, precipitation of driest month; BIO3, isothermality; BIO4, temperature seasonality; BIO5, maximum temperature of warmest month; Elev, elevation; TPI, topographic position index.

For model building and calibration, 107 presence records were selected after spatial and environmental filtering (Figure 2, Appendix S1). The bioclimatic predictors selected for the modelling process based on VIF analysis were BIO3 (isothermality), BIO4 (temperature seasonality), BIO5 (maximum temperature of the warmest month), BIO12 (annual precipitation) and BIO14 (precipitation of the driest month); topographic variables were elevation, slope and topographic position index and land cover was forest variable (Table 1). When the variables affecting the model are analysed, it is seen that the bioclimatic variables (precipitation of the driest month (BIO14), seasonality of temperature (BIO4) and annual precipitation (BIO12)) were the most effective factors, while topography and land cover variables contributed very little (Figure 3). Considering that the studied species is a saproxylic beetle species and spends its pre‐adult stages in decaying logs, it is not surprising that rainfall and temperature are the most effective factors. This species, which emerges as an adult in early summer, will mate and lay eggs during the driest periods of summer. In this context, the amount of rainfall in the driest month will have an effect on the eggs to be laid on moist logs. It is reasonable to posit that the forest variable will contribute more in this context. However, it should be noted that this variable provides the percentage of wooded area. The health of the trees is likely to be of greater importance for the species in question and will undoubtedly exert a greater influence on the distribution of the species than whether the wooded areas are dense or not.

### 3.2 Model Performance

After the preliminary evaluation for Maxent, the final model was developed using different FCs and RM for each set (set 1: LQ‐1.3, set 2: H‐1.2, set 3: LQHP‐2.9, and set 4: H‐1.7) (Appendix S3). Among the single models, models with an AUC score higher than 0.80 were included in the ensemble model (set 1: 120 single models, set 2: 131 single models, set 3: 132 single models and set 4: 130 single models). The SRE algorithm did not show an AUC value above 80% in any of its iterations and could not be included in any ensemble model. GBM, GLM and RF algorithms were the best performing algorithms, each contributing a total of 63 single models for the four sets. The Ensemble model obtained with the ‘wmean’ algorithm reported high AUC values (set 1 = 0.975, set 2 = 0.982, set 3 = 0.975, set 4 = 0.982).

### 3.3 Suitable Habitats Predicted for Current Conditions

**FIGURE 4:**
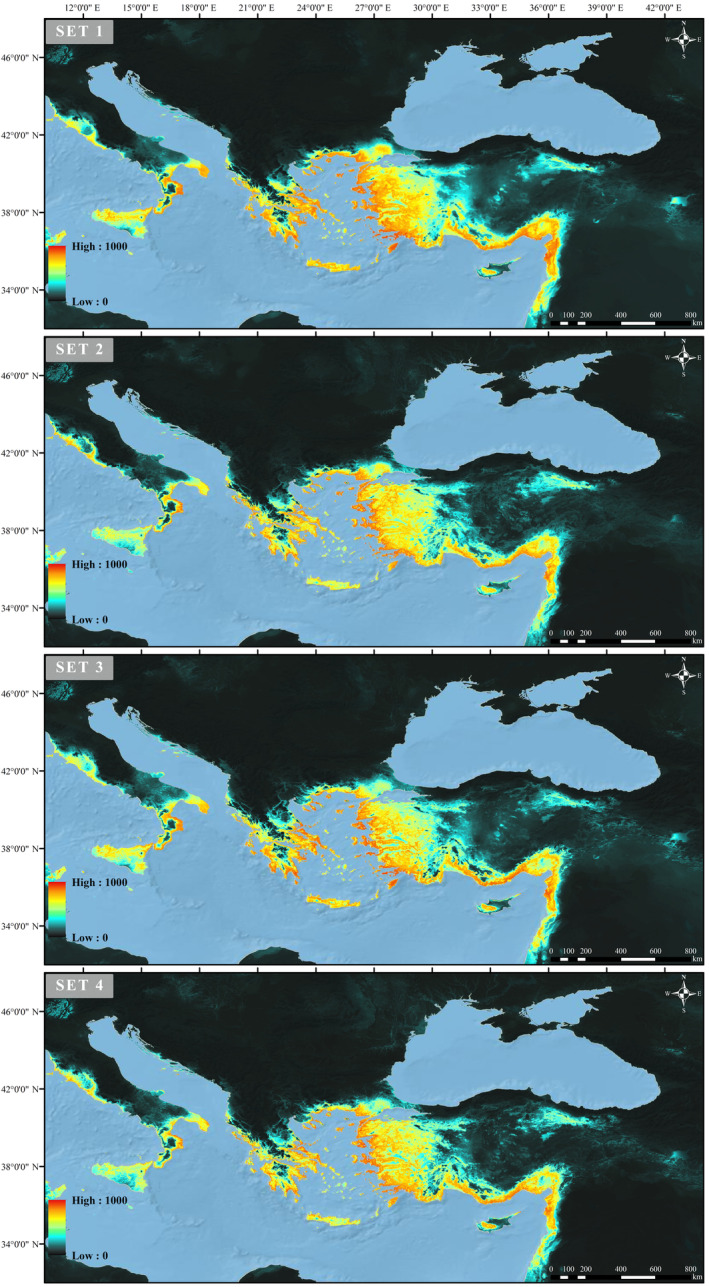
Suitable habitats for *Chalcophora detrita* under current conditions with four different sets of predictors.

The model results show that, under current conditions, suitable areas are relatively consistent with the known distribution of the species. Areas where the species is known to occur, but presence records are either absent (e.g., Cyprus) or relatively poor (e.g., Italy) were also predicted by the models. In the Balkans (e.g., Bulgaria), where the species is known to occur, no coordinate presence records could be obtained, and the models did not show a bioclimatic suitability. The models also showed the Central Black Sea Region of Türkiye and Sicily Italy as bioclimatic suitable areas, but no data for the presence of the species were found from this region (Figure 4).

### 3.4 Suitable Habitats Predicted for Future Conditions

**FIGURE 5:**
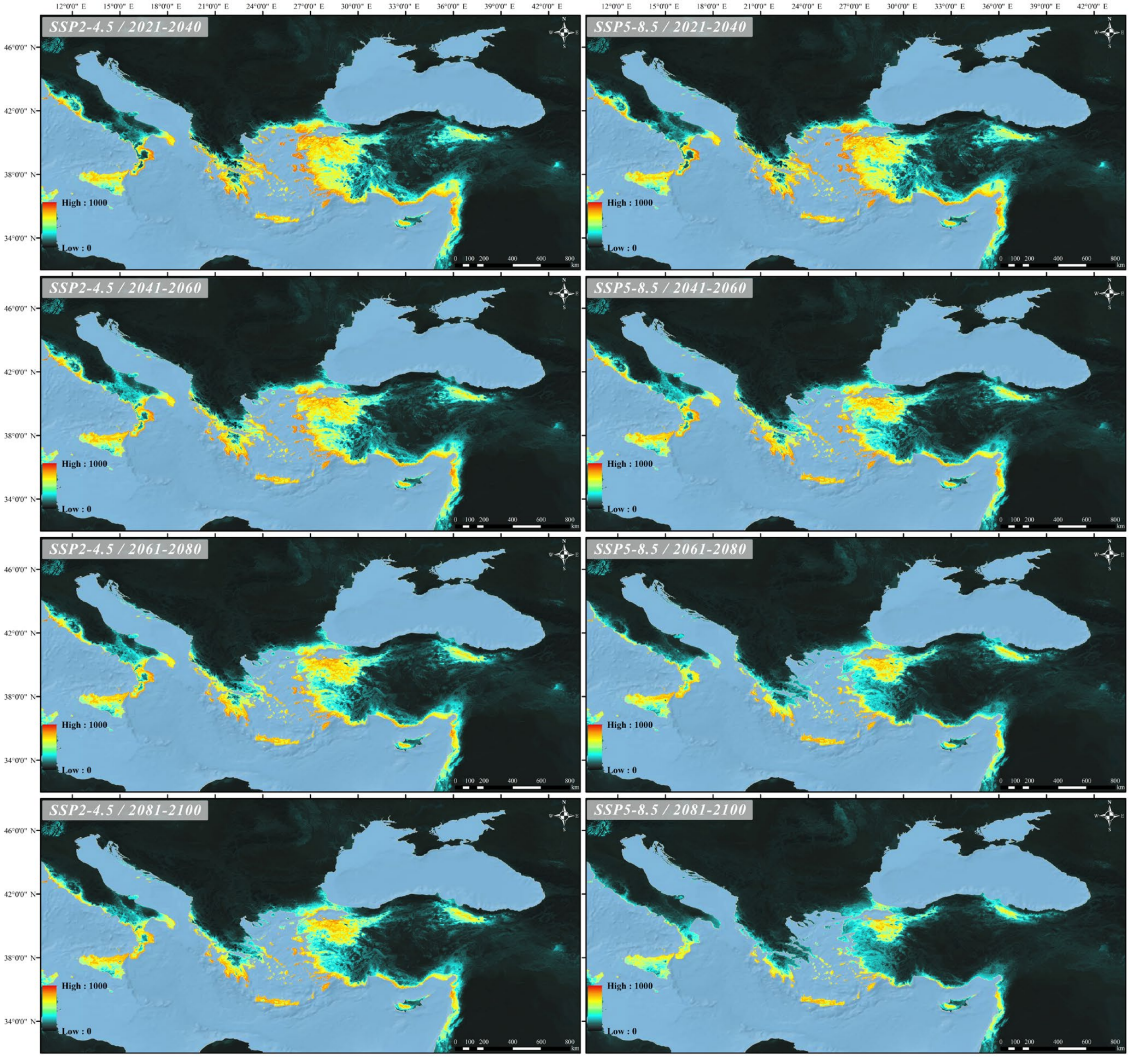
Potential suitable area of *C. detrita* under future climate conditions.

**FIGURE 6:**
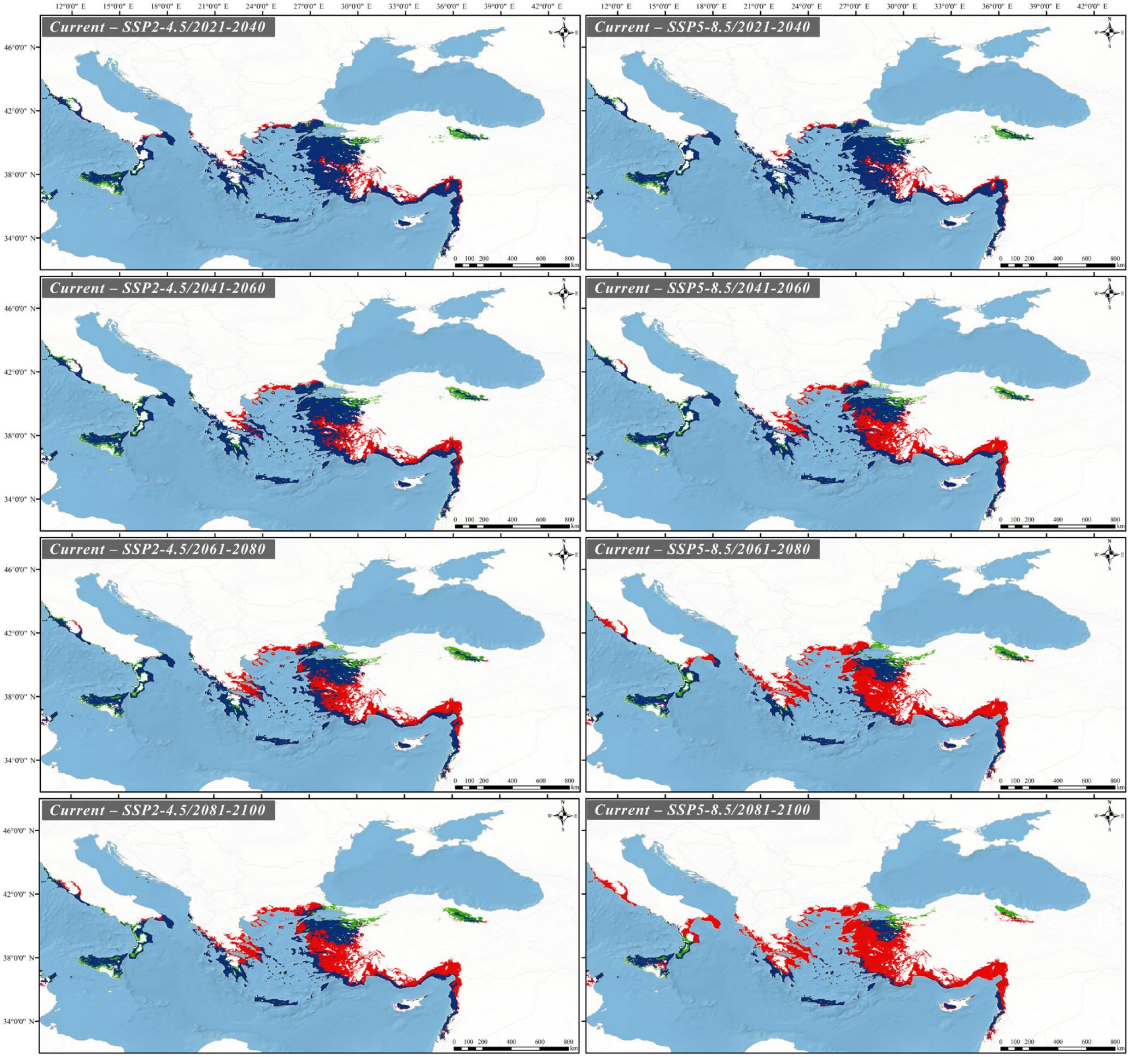
Range change in predicted suitable habitats for *C. detrita*.

Future projections were carried out under two scenarios (SSP2‐4.5, relatively optimistic and SSP5‐8.5, most pessimistic). In both scenarios, when comparing the species bioclimatic suitable areas with the current projection, it has been observed that these areas have significantly decreased gradually each year (Table 2). According to the scenarios, the decline of suitable areas in Italy, the Aegean islands, Cyprus and Aegean coast of Türkiye is less than in other regions. According to 2081–2100 period of the most pessimistic scenario, the high mountains of the Marmara Region and its vicinity (Mountains of Alaçam, Simav, Eğrigöz, Umurlar, Kaz, Uludağ, etc.), the southern coasts of the Peloponnese and the Aegean islands in Greece, the southern part of Italy (Calabria region) and Cyprus (Troodos Mountains) are predicted to remain as suitable areas (Figures 5 and 6).

## 4 Discussion

The awareness regarding the significance of deadwood in forest ecosystems continues to increase. Deadwood serves a crucial role in carbon sequestration, nutrient provision and water retention (Lachat et al. 2013). Consequently, awareness of the importance of saproxylic biodiversity has begun to proportionally rise. In this study, current and future bioclimatic suitable habitats of *Chalcophora detrita*, a saproxylic species, were predicted for the first time with ENM.

Saproxylic beetles are a highly threatened functional group of forest ecosystems (Lachat et al. 2012). Della Roca et al. (2019) showed that according to the three different future scenarios, climate change will result in the reduction of rare saproxylic richness towards limited areas along the Alps and the Apennines in 2070. Also, according to Carpaneto et al. (2015) the IUCN category (Italy) for *Chalcophora detrita detrita* is endangered. Nieto and Alexander (2010) demonstrated that 10.7% of saproxylic insect species in Europe were under the threat of extinction. Moreover, in the study conducted by Avgın, Dertli, and Barsevskis (2014), 151 insect species included in the European Red List of Saproxylic Beetles live in Türkiye. When considering the last study in conjunction with research conducted in Italy, it indicates that the Mediterranean coastline deserves further investigation in terms of the responses of saproxylic insects to climate change.

The results obtained in this study were achieved solely using the ENM method. Therefore, when interpreting the results, certain factors not accounted for by the method should be considered. For instance, one of the important issues that needs to be discussed is the observation that the populations of this species, whose role in the forest ecosystem is not yet fully understood, are adversely affected by anthropogenic factors. Habitat destruction, inappropriate forest management and forest fires can be considered as the most important of these factors. An example of inappropriate forest management is the use of pheromone traps. When this method of controlling forest pests is applied in an unplanned manner, the result is damage to other species rather than the target pests (Etxebeste, Lencina, and Pajares 2013; Bracalini et al. 2021; Bracalini, Florenzano, and Panzavolta 2024). In our independent field studies conducted in the Inner Western Anatolia region of Türkiye in 2019, 2021 and 2022, we detected a significant number of *Chalcophora detrita* individuals in pheromone traps, despite them not being the primary target. Our observations indicate that these traps are set in large numbers at very short distances from each other and over very large areas.

Forest fires, another factor affecting the distribution of the species, have become a natural evolutionary process, especially in forests with plant populations adapted to burning. It can be easily predicted that the frequency of forest fires will affect the distribution of *Chalcophora detrita* populations, which feeds on *Pinus* species exposed to fire. According to the European Forest Fire Information System (EFFIS), between 2006 and 2023, examining the data on forest fires in the distribution areas of this species worldwide reveals that, on average annually, 56,673.17 ha in Italy, 50,783.28 ha in Greece, 10,578.28 ha in Bulgaria, 44,197.61 ha in Türkiye, 1728.06 ha in Cyprus, 21,805.06 ha in Syria, 510.11 ha in Lebanon and 1606.17 ha in Israel have burned. Similarly, examining the same data reveals that, on average annually, 290.28 forest fires in Italy, 56.5 in Greece, 29.72 in Bulgaria, 124.39 in Turkey, 6.06 in Cyprus, 32.22 in Syria, 4.94 in Lebanon and 4.78 in Israel have occurred (access date: 6.7.2024).

In addition to all other potential factors, the results of the ENM should be evaluated by considering biotic factors such as predator pressure, dispersal, feeding habits, life cycle, reproduction rate and pathogens. In this context, every study that sheds light on the biology of the species, in addition to taxonomic studies, is valuable. As mentioned below, for instance, the ecological role of the species is also a subject of debate.

Besides being saproxylic insects, another characteristic that makes *Chalcophora* species intriguing in terms of the consequences of climate change is their economic significance. Indeed, many Buprestidae species can be considered as secondary pests in terms of their feeding habits (Salle, Nageleisen, and Lieutier 2014). Although it was stated in the study of Bark and Wood Boring Insects in Trees in Europe by Lieutier et al. (2004) that global warming would make forests vulnerable to insect attacks, the genus *Chalcophora* was not evaluated under the category of ‘Buprestidae Species that Damage Trees’ in the same study. In fact, the economic importance of species belonging to the genus *Chalcophora* is controversial. Maier and Ivie (2013) mentioned the economic importance of this genus by considering the biogeographical conditions of North America. According to Barbosa and Wagner (1989), two *Chalcophora* species native to North America attack the wood pulp, rendering the log worthless, and can cause serious damage. Some of the authors mentioned *Chalcophora detrita* as the pest of the *Pinus* species (Akçay and Yalçın 2019; Yalçın et al. 2019; Pantelas 1986). As a result, like other *Chalcophora* species, *Chalcophora detrita* is a challenging organism that will make climate change studies more interesting both as a forest pest and as a saproxylic insect species.

Given this controversial ecological role of *Chalcophora detrita*, future scenarios should consider not only the loss but also the gains of suitable areas. In both scenarios (SSP2‐4.5, relatively optimistic and SSP5‐8.5, most pessimistic), when comparing the species bioclimatic suitable areas with the current projection, although the rate of lost areas is higher, it has been observed that new suitable areas have been gained (Table 1). Studies that take into account biotic factors such as the presence of dead and rotten trees, competition, behaviour, feeding habits and life cycle will provide healthier results on issues such as gaining new suitable habitats.

To accurately interpret a species' ecological role and its relationship with other elements in its habitat, as well as to understand how realistic predictions about the species' future are, it is necessary to consider the geological history of its distribution area. It is believed that xylophagous buprestids first appeared in the Middle Jurassic but became very widespread towards the end of the Lower Cretaceous and remained one of the most common beetle groups until the Eocene (Ponomarenko 2003). An examination of the geological history of the species' distribution area reveals the significance of the Eocene and subsequent periods. During this time, the Tethys Ocean began to close, accompanied by various tectonic movements. This process led to the formation of the current geological structure of Anatolia and the Aegean Region (Ricou 1996). The Miocene epoch, commencing approximately 23 million years ago, was characterised by significant tectonic activities in the Aegean Region. The collision between the African and Eurasian plates induced stretching and expansion in the Aegean Sea. Consequently, the accelerated expansion during the Middle Miocene, around 15 million years ago, resulted in the formation of extensive graben systems, which were subsequently inundated due to rising sea levels (Sakellariou et al. 2021). Geographical isolation between the populations of this species on both sides of the Aegean Sea likely began during this period. This could explain why the current populations in Anatolia and Greece are classified into two distinct subspecies: *C. detrita detrita* and *C. detrita marani*. The observation that populations on the Aegean Islands belong to the same subspecies (*C. detrita marani*) can be attributed to the decreased sea levels during glacial periods (Papanikolaou 2021), which facilitated the formation of land bridges between the islands and the mainland. On the other hand, the dispersal capability of the species is not fully known, and the possibility that it may have flown across the distance between the islands cannot be ruled out. This possibility underscores the importance of understanding biotic factors in ENM studies.

There are two different perspectives regarding Cyprus's connection to the mainland. The first posits that after Cyprus detached from Africa, it never reconnected with the mainland and has remained isolated. The second perspective suggests that the island established land connections with Southern Anatolia or Syria at various times throughout its geological history (for detailed references, see Hadjisterkotis, Masala, and Reese 2000). In the first scenario, it can be inferred that the species' populations distributed to the island while it was still connected to the mainland and subsequently became isolated over time following the separation from the mainland. However, due to the absence of the distribution of the species *Chalcophora detrita* in North Africa and the lack of any evidence supporting its historical presence in this region, the validity of this scenario can be disregarded. In the second scenario, land bridges formed between Anatolia and Cyprus, facilitating the distribution of the species' populations to Cyprus. Once these land bridges disappeared, these populations became isolated on the island, eventually leading to the emergence of the subspecies *C. detrita margotana*, which is currently found in Cyprus. However, it should be noted that the differences between these subspecies are based on morphological characteristics, and genetic studies on this species are very limited. Future genetic studies involving different populations of the species are expected to shed much more light on this situation. Indeed, data obtained from previous studies on the refuge role and genetic diversity of Anatolia have been highly functional in this regard so far. For example, in their study, Çıplak et al. (2015) suggested that cold‐adapted lineages with low dispersal capability may not have colonised regions outside the Anatolian refuge during interglacial periods. Additionally, they indicated that the distribution ranges of invertebrate populations might be limited, but this does not necessarily imply that their effective population sizes are small. Notably, these conclusions could challenge the basis of our discussion regarding the potential deterministic relationship between the dispersal ability of Chalcophora detrita and its distribution ranges, because the importance of Anatolia's role as a refuge comes to the forefront, and the reproductive mechanisms in invertebrate populations can vary. In addition to all this, when considering inferences regarding Anatolia's role as a ‘refuge within a refuge’ during the Last Interglacial Period (Perktaş et al. 2015), the foundation of insect biodiversity in this region can be better understood. Also in their phylogeographic study on the origin of the meadow grasshopper species *Chorthippus parallelus*, Korkmaz et al. (2014) tested the nature of the Turkish Straits system as a barrier to distribution and identified the Straits as a weak barrier. This finding casts doubt on the data suggesting that no transition has been observed between populations of the two subspecies, Chalcophora detrita detrita and Chalcophora detrita marani, in the different geographical regions where they are distributed. One of the interesting results of our modellings is that although it has been observed that the suitable habitats of *C. detrita marani* and *C. detrita detrita* subspecies have decreased, the *C. detrita margotana* subspecies, which is distributed in Cyprus, will not be affected much in the future. This may be due to the fact that the subspecies in question distributes on an island. However, it should be noted that the suitable habitats indicated by the model results are the forested areas in the Troodos Mountains, which are at risk of negative impacts from anthropogenic factors such as deforestation for agriculture, forest fires, and inappropriate land use. Also, unlike the populations of *C. detrita marani* and *C. detrita detrita* subspecies live on the mainland, our study indicates that the populations of these subspecies on the Aegean islands are not affected much. Although the *C. detrita marani* subspecies in Greece has retreated to the south, its suitable habitat is preserved in Crete. In addition, in our study, we predict that the habitats of the *C. detrita detrita* subspecies in Türkiye will be fragmented (Figures 4 and 5).

The ENM method we used in our study has long been used in research on saproxylic organisms, species of economic importance, vectors and other living organisms with various ecological roles. The use of this method is becoming increasingly widespread and easier in parallel with technological developments (Costa and Peterson 2011). However, this method has its own disadvantages, and the results should be interpreted with caution.

The distribution of suitable habitats for *Chalcophora detrita*, which we modelled in our study, should be interpreted together with the physiological and behavioural studies to be conducted on this species. In this context, we believe that biotic factors are important and the physiological studies at the species level should keep up with the pace of taxonomic publications. In addition, we believe that ENM studies on saproxylic species to be conducted in the near future will provide very important data to protect forest health.

## Author Contributions

**Arif Duyar:** investigation (equal), writing – original draft (equal). **Muhammed Arif Demir:** investigation (equal), writing – original draft (equal). **Mahmut Kabalak:** conceptualization (equal), supervision (equal), writing – original draft (equal), writing – review and editing (equal).

## Conflicts of Interest

The authors declare no conflicts of interest.

## Supporting information


Appendix S1

Appendix S2

Appendix S3

## Data Availability

The data that support the findings of this study are available in the Appendices [Link], [Link], [Link] of this article.
