# Supplementary material for: Prediction of Current and Future Distributions of Chalcophora detrita (Coleoptera: Buprestidae) Under Climate Change Scenarios
Source: Ecol Evol. 2025 Jan 16;15(1):e70693. doi: 10.1002/ece3.70693 (PMC11739133; doi:10.1002/ece3.70693)

**Supplemental Information for:**

**Prediction of current and future distributions of *Chalcophora detrita* (Coleoptera: Buprestidae) under climate change scenarios**

Arif Duyar^1,2^ (ORCID: <https://orcid.org/0009-0002-3931-4448>), Muhammed Arif Demir^1^ (ORCID: <https://orcid.org/0000-0001-7141-0814>), Mahmut Kabalak^3,4*^ (ORCID: <https://orcid.org/0000-0001-6073-2551>)

^1^Hacettepe University, Graduate School of Science and Engineering, Department of Applied Biology, Ankara, Türkiye.

^2^The Scientific and Technological Research Council of Türkiye (TÜBİTAK)

^3^Hacettepe University, Faculty of Science, Biology Department, Ankara, Türkiye.

^4^Hacettepe University, Biological Diversity Research and Application Center, Beytepe, Ankara, Türkiye

***Corresponding author:** Mahmut Kabalak, [mahmut@hacettepe.edu.tr](mailto:mahmut@hacettepe.edu.tr)

**Supplementary Figure S2.** Impact of simulation parameters on M areas obtained from simulations under current conditions for *Chalcophora detrita*. The occurrence records used to initiate the simulations are indicated by black dots. The modified simulation parameters (Number of dispersal events and Kernel SD) and the highest AUC value (with RM and FC values) obtained from Maxent models using these M areas along with current projections are presented. The simulation outlined in red represents the one used in the final models.


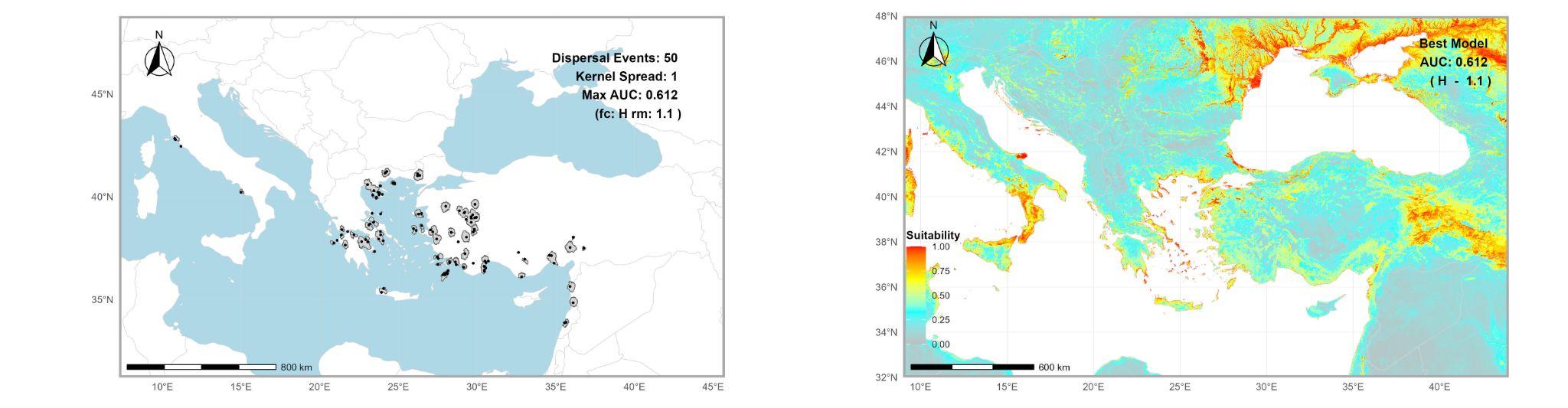


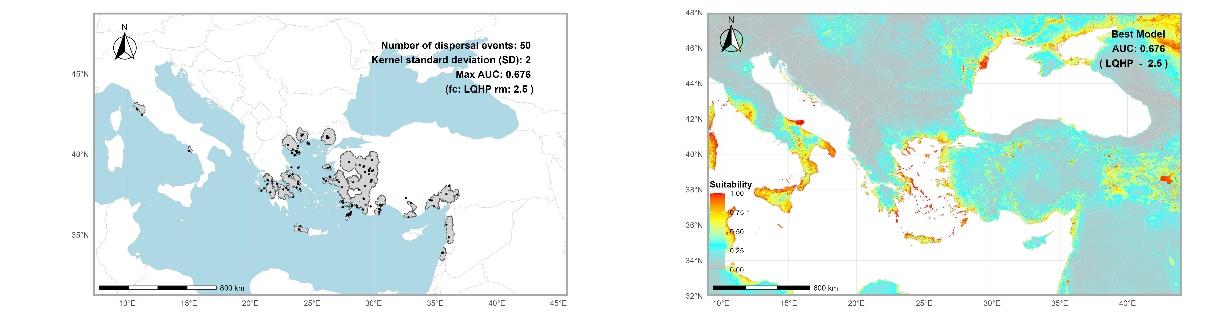

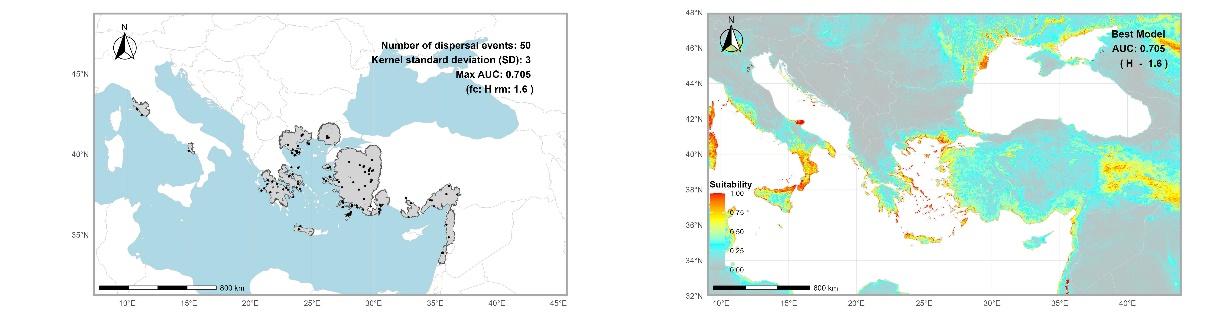


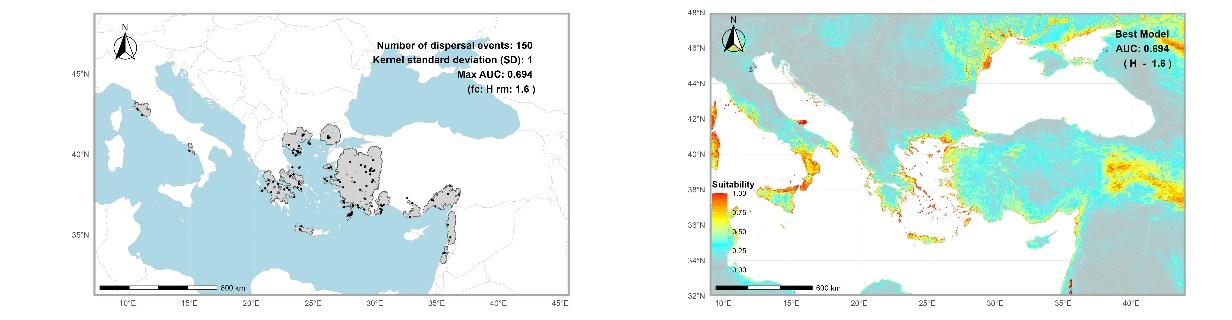

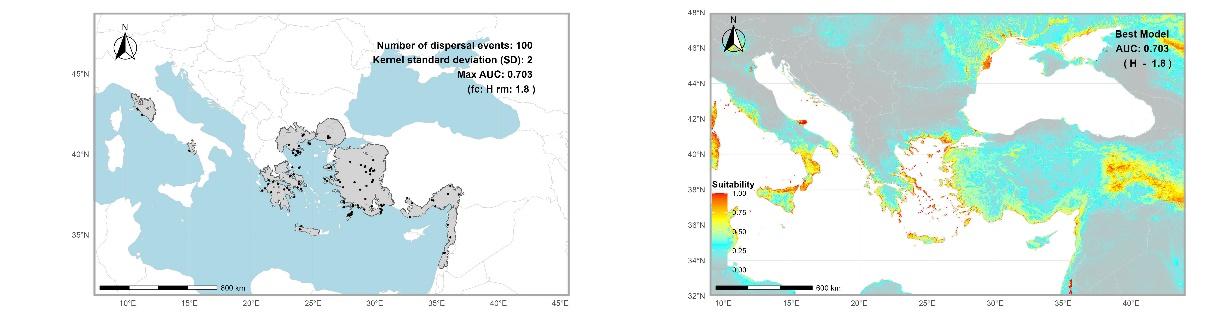

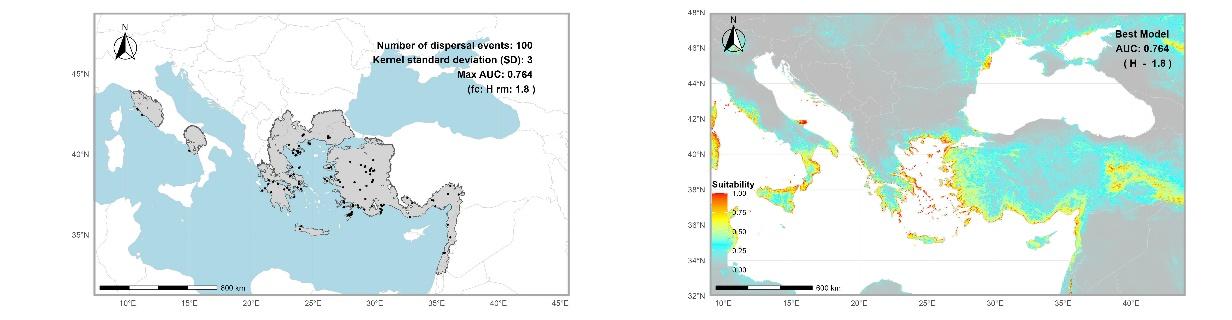


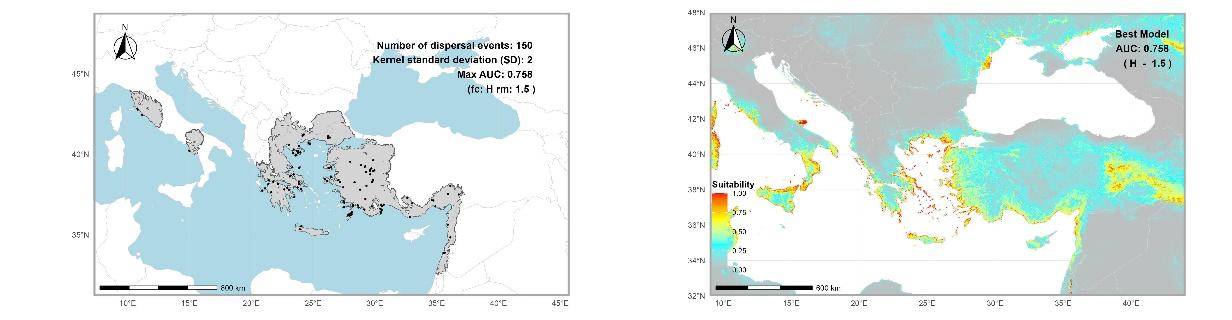


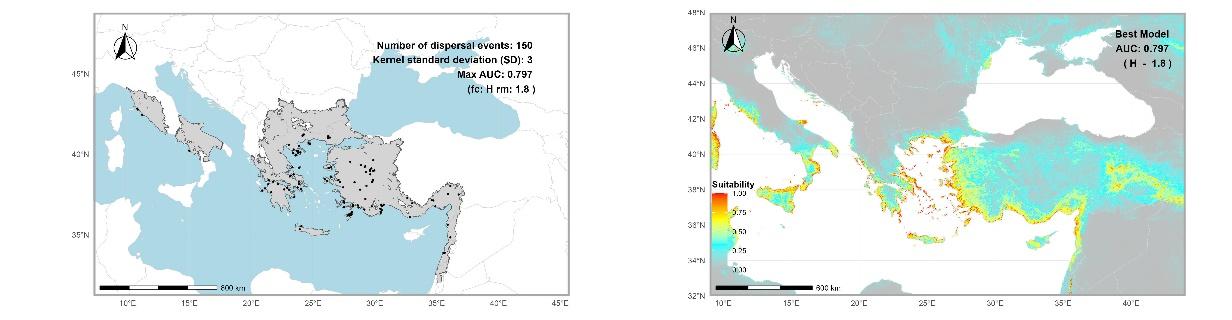


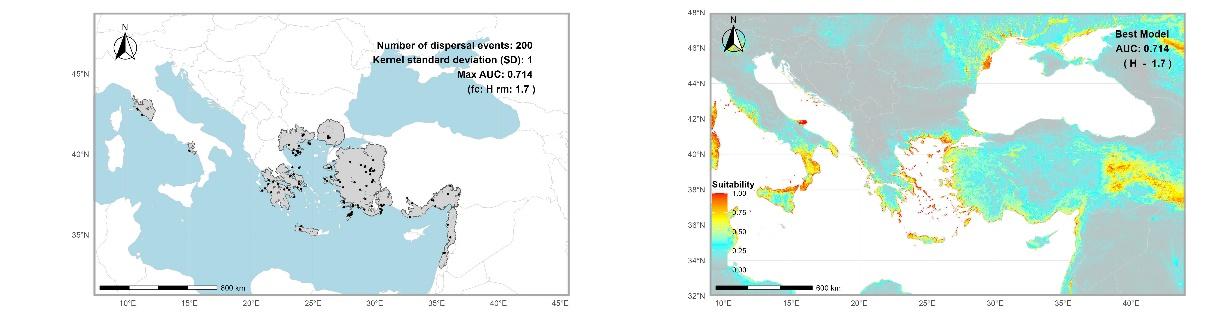

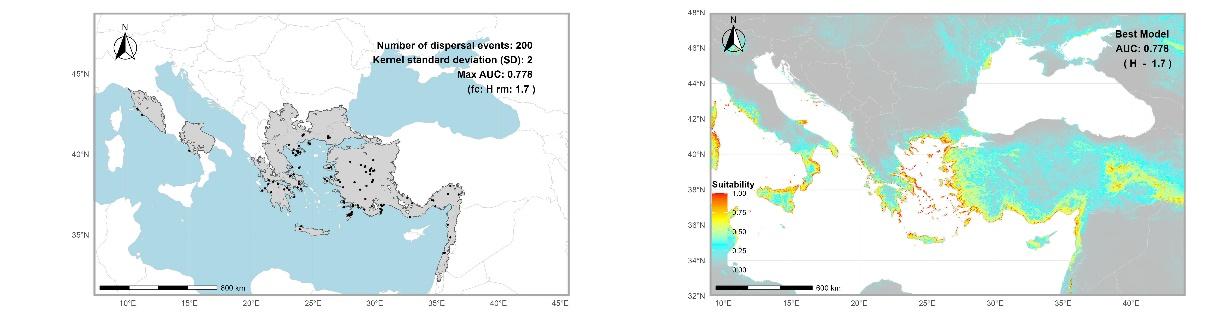


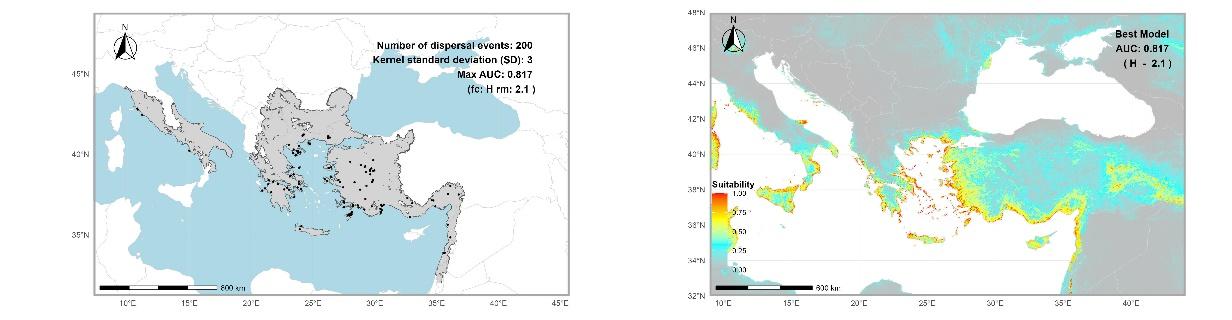


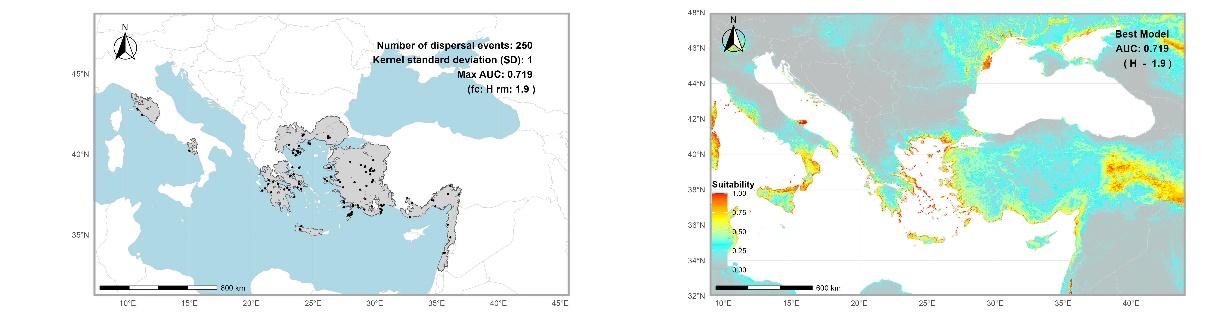

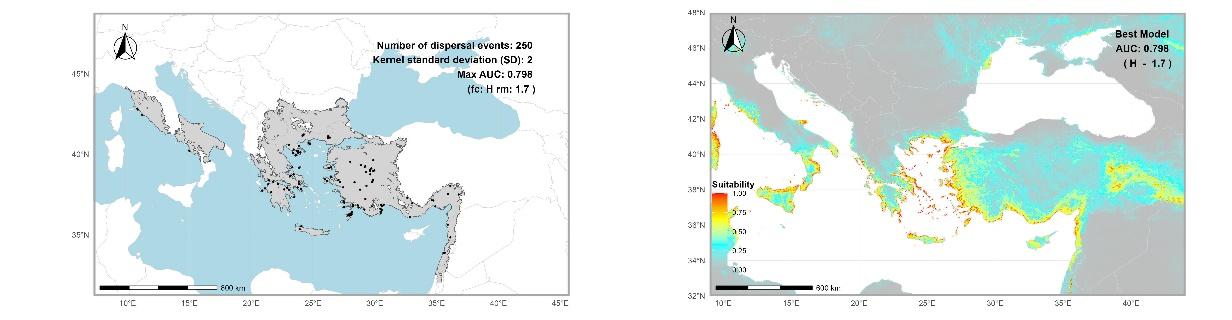

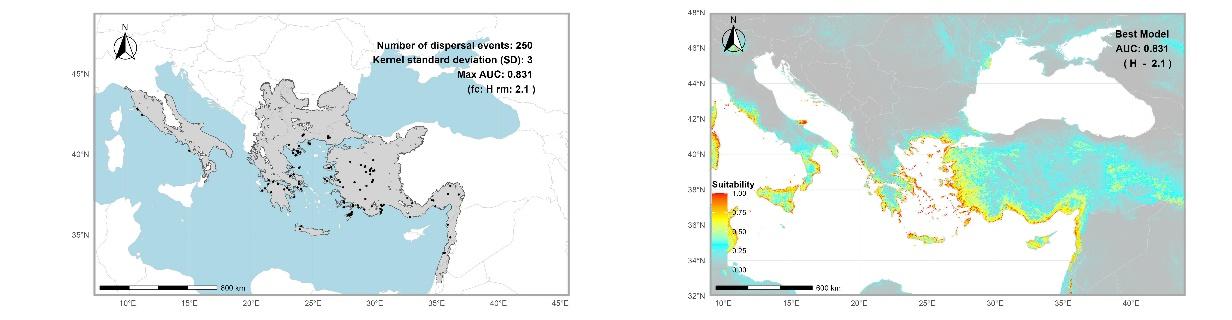


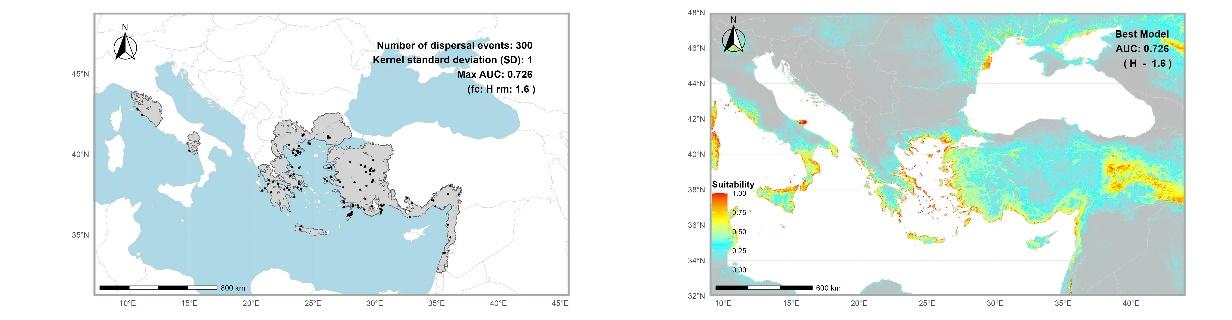

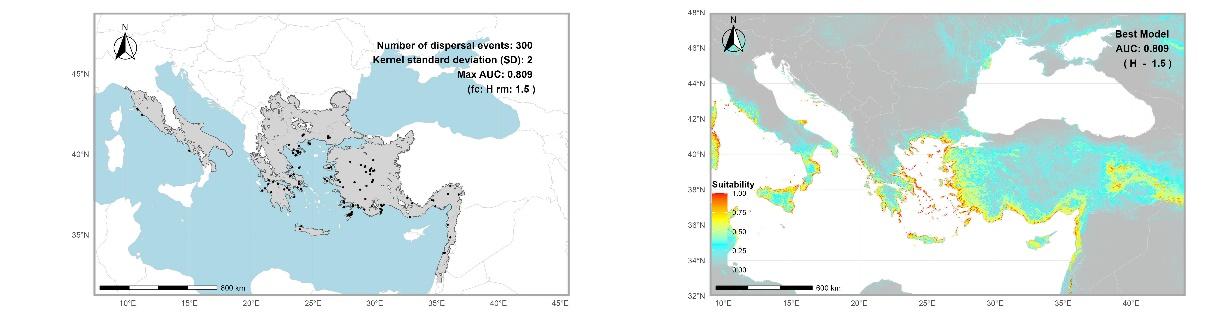

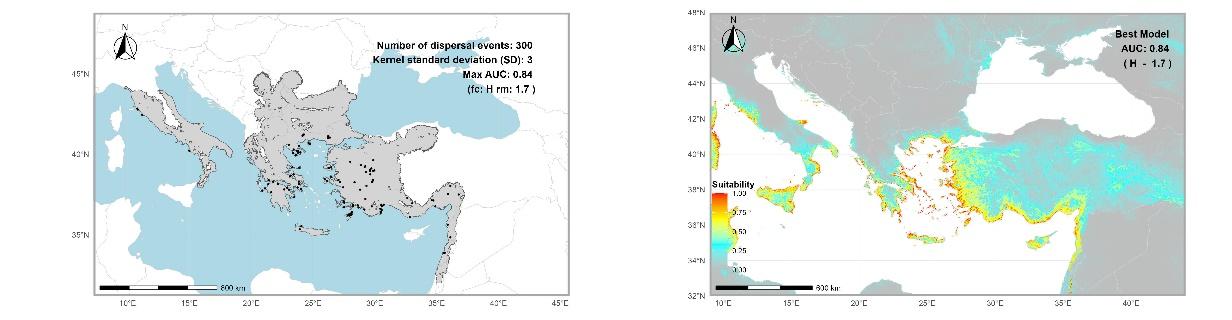


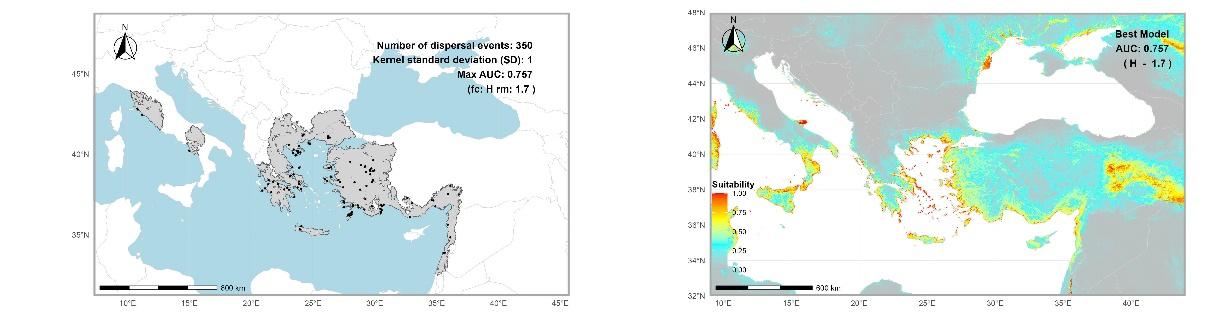

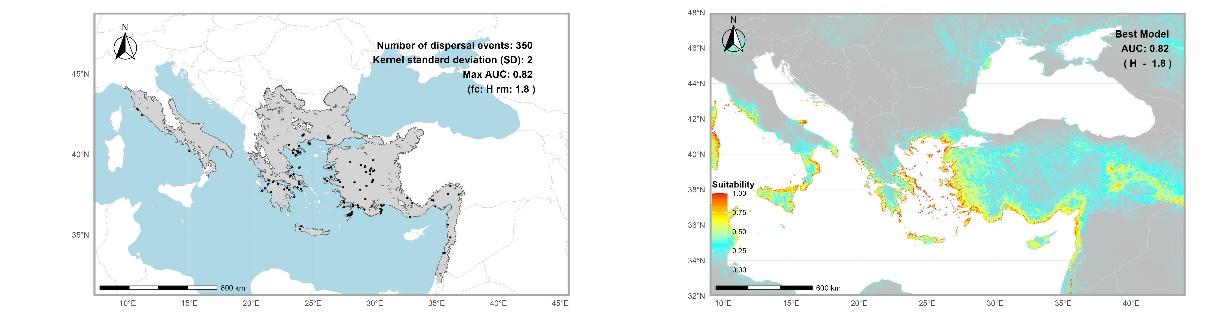

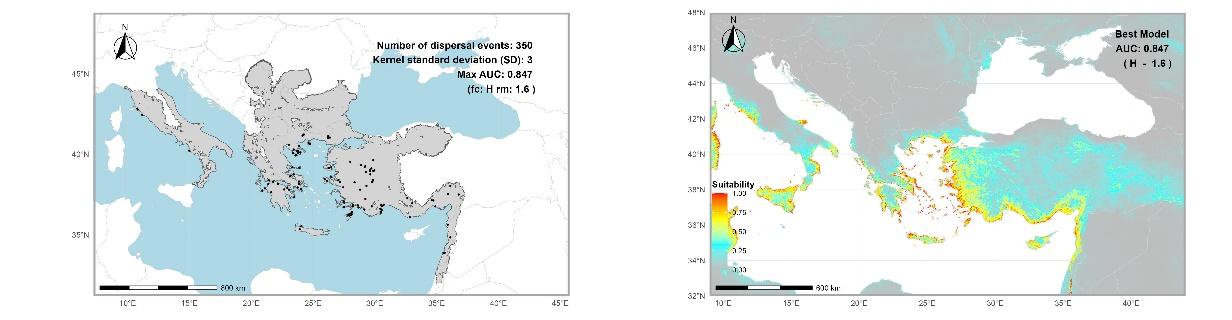

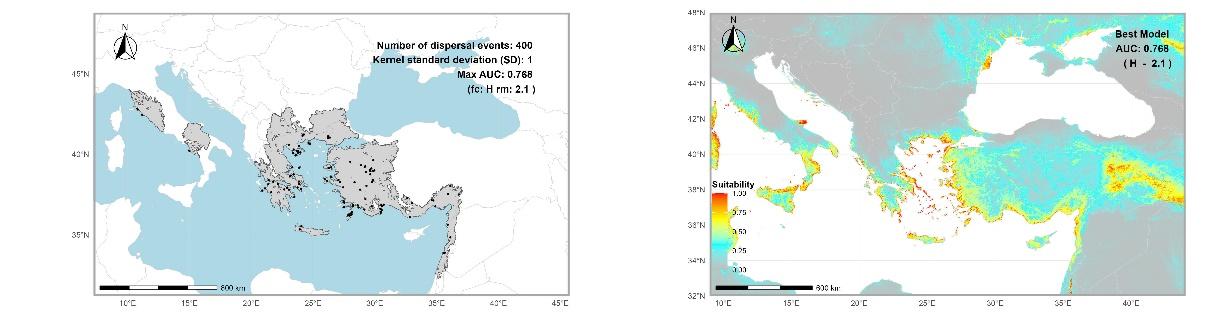

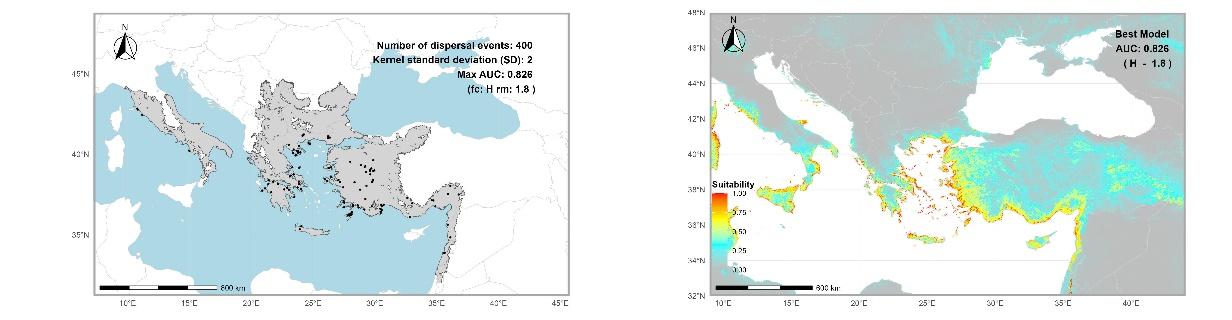

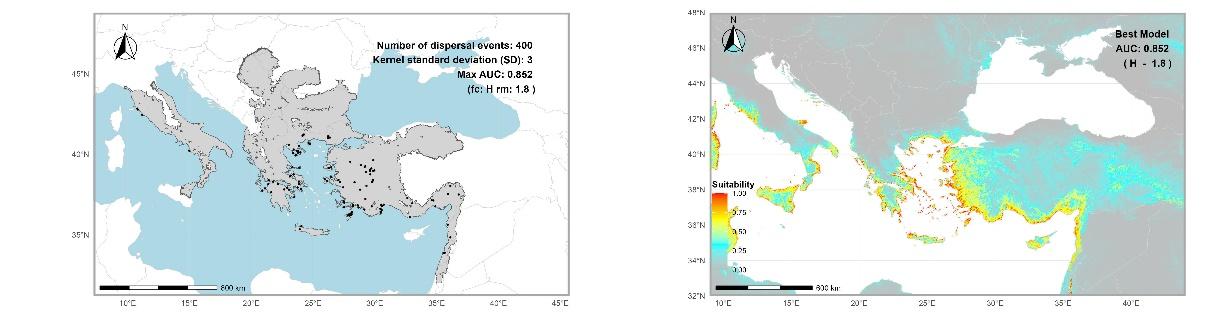

Supplement: Supplementary file 2 — Appendix S2 [file ECE3-15-e70693-s002.docx]
